# Resettlement and spatial injustices: governance and water access challenges for IDPs in Hargeisa, Somaliland

**DOI:** 10.1039/d6ew00002a

**Published:** 2026-10-06

**Authors:** Ayan Hujaleh, Taimaz Larimian, Susie Goodall

**Affiliations:** a School of Architecture, Building and Civil Engineering, Loughborough University Loughborough UK a.hujaleh@lboro.ac.uk

## Abstract

This qualitative study explored how government and NGO interventions address the water needs of internally displaced persons (IDPs) in Hargeisa, Somaliland. A total of 30 interviews were conducted with those providing support, informal service providers and IDP committee leaders. This study presents four overarching insights reflecting important aspects of the water services to IDPs: (i) resettlement secures IDPs with land but undermines their access to water service, (ii) organisation centralisation and disconnection weakening responses, (iii) formal water expansion disrupts livelihoods, and (iv) the limited autonomy of committee leaders. The findings underscore systemic gaps in Hargeisa's water infrastructure and highlight the barriers and governance dynamics undermining water access for IDPs from a multi-stakeholder perspective.

## Abstract 2

This qualitative study explored how government and NGO interventions address the water needs of internally displaced persons (IDPs) in Hargeisa, Somaliland.

## Graphical abstract

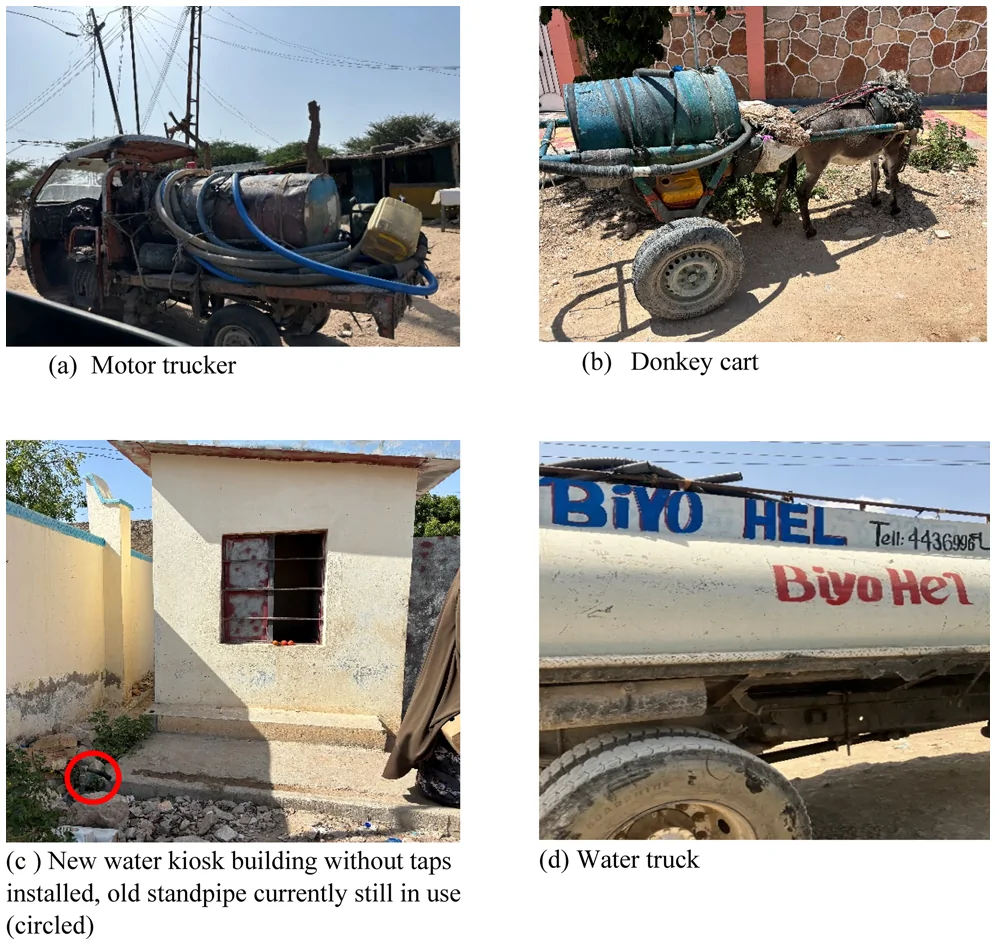

**Water impact**

Population displacement places growing pressure on urban water systems. This study examines how governance gaps undermine water access for IDPs and situates water access within spatial justice, showing how repeated displacement intersects with inequalities. Findings reveal that resettled IDPs face greater challenges to livelihoods and water access, highlighting systemic infrastructure gaps and offering practical insights for policymakers and humanitarian actors.

## 1. Introduction

The last decade has witnessed a rapid rise in population displacement, reaching record-high numbers of internally displaced persons (IDPs), with a total of 83.4 million at the end of 2024.^1^ The United Nations defines IDPs as people who are forced to leave their homes to avoid the effects of armed conflict, situations of generalised violence, violations of human rights or natural or human-made disasters, without crossing international borders.^2^

As population displacement increases, so does the strain on public services, with access to water emerging as one of the most persistent challenges.^3^ In several displacement settings, limited resources and poor infrastructure makes water inaccessible for many.^4^ Non-governmental organisations (NGOs) and governments play a critical role in providing assistance to affected populations,^5,6^ but long-term sustainable solutions for water access remain limited,^7^ particularly with reduced funding across the donor landscape.^8^

The provision of services and assistance to IDPs is the responsibility of the national authorities of the countries they reside in according to Principle 3 of the Guiding Principles on Internal Displacement.^9^ This responsibility includes ensuring the IDPs have safe access to essential food, basic shelter and housing, appropriate clothing, essential medical services, sanitation and potable water as affirmed by Principle 18. However, in many contexts, the growing scale of displacement has stretched the capacity of governments to respond.^10^ This is especially evident across conflict-affected African countries, including the Democratic Republic of the Congo (DRC) and Sudan, where displacement of more than 20 million people has placed severe pressure on government capacity, humanitarian infrastructure and the provision of essential services.^11^ Support from governments is often fragmented and lacks effective coordination with NGOs, resulting in duplicate works, often targeting the same communities and leaving others without adequate support.^12^

Somalia illustrates these dynamics acutely, with resettlement programmes increasingly used by the government to move IPDs out of informal settlements and into more permanent arrangements. Yet these efforts raise complex questions about land rights and long-term integration, as well as the provision of basic services. IDPs are resettled to the city's outskirts, often in remote and underdeveloped areas, far from schools, markets, healthcare facilities, and previous sources of employment. As a result, these schemes can worsen water accessibility whilst aiming to address shelter needs.^13^ This research focuses on examining the processes, challenges, and outcomes of these resettlement initiatives, with particular attention to the perspectives and agency of displaced communities themselves, and the coordination or lack thereof among institutional actors involved in these efforts.

Despite the increasing attention on water provision in displacement settings, there remains limited research examining how interventions align with the lived experiences and needs of IDPs themselves.^14^ Previous studies have predominantly focused on the technical and logistical challenges of water provision in displacement settings; these include water quality,^15^ water treatment,^16^ funding constraints^17^ and institutional capacity.^18^ Many of these studies overlook the views of various stakeholders.^19^ Fewer have examined how consequences of resettlement unfold across social, economic and service delivery domains together.

This research aimed to explore the views of three stakeholder groups: those providing support to IDPs (including NGOs, not-for-profits (NFPs) and the Government), informal service providers and IDP committee leaders on: what water support (potable water provision under Principle 18 of the UN Guiding Principles) is being provided to IDPs and to what extent does this align with their self-identified needs? What unintended consequences across the social, economic and service- delivery domains have resulted from the resettlement of IDPs and how are these challenges being addressed by humanitarian and government actors?

This paper is divided into five sections. Section 2 provides the theoretical background, outlining the key concepts and frameworks. Section 3 presents the results of the qualitative analysis, highlighting four key themes that emerged from the data. Section 4 analyses these themes in relation to the existing literature and highlights the broader implications. Section 5 concludes with a summary of the main findings, discusses the limitations of the study and offers recommendations for future research.

## 2. Theoretical framework

Spatial justice refers to the equitable distribution of infrastructure, services, political power, and environmental goods across different geographical areas.^20^ The concept of spatial justice has been widely explored by scholars; however, the term ‘spatial justice’ only gained widespread use in the early 2000s largely through the work of Soja.^21^ Marcuse^22^ recognised the interconnection between space and social relations. He outlines five propositions showing that spatial injustice is rooted in broader social, political, and economic inequalities. Other scholars have underscored that justice involves more than the equitable distribution of resources and services but also entails participation in shaping the spaces people live.^23,24^

For this study, spatial justice is understood through both distributive and procedural dimensions. The distributive dimension concerns how essential infrastructure and services are spatially allocated, including whether resettled IDPs experience unequal access because of their peripheral location. The procedural dimension concerns the extent to which displaced communities are able to participate in decisions about resettlement, infrastructure and service provision. Together, these dimensions provide a framework for examining not only where water services are available, but also how decisions about their provision are made and whose priorities are represented.^25–27^

Past research has examined water and infrastructure provision specifically in the context of displacement. In Germany, following the large-scale arrival of displaced persons in 2016, researchers examined how host communities perceived permanent *versus* temporary infrastructure solutions for extending water services, and how public support for utility expansion was shaped by place attachment and demographic factors.^28^ Gnowa *et al.*^29^ similarly showed that resilient infrastructure planning in refugee and IDP settlements is distinct from conventional planning, centring on rapidly deployable, often temporary solutions under conditions of institutional uncertainty and resource constraint.

In Beira, Mozambique, inequalities in household water and sanitation access persist across low-income peri-urban neighbourhoods, with service reliability and satisfaction declining as distance from the main water distribution network increases.^30^ This spatial dimension of exclusion is also evident in broader reviews of WASH policy in informal settlements, which identify economic, spatial, social, institutional, political, and informational barriers to service provision. Spatial barriers arise from the location and physical structure of informal settlements, shaping residents' access to essential services.^31^

However, few studies have applied a spatial justice lens explicitly to water infrastructure planning in marginalised displacement contexts. This gap is particularly relevant to Somalia, where resettlement sites are typically located on the urban periphery and may be disconnected from the water infrastructure.^13^ Understanding how these spatial and institutional disconnections shape water access is therefore critical to assessing whether resettlement addresses or inadvertently reinforces existing inequalities.

Displacement should be understood as an evolving process rather than a one-off occurrence. Many IDPs face multiple displacements rather than a single event of physical relocation. For example, an IDP may initially flee their homes due to conflict, later cross a border to become a refugee, then return as a returnee, often returning to unstable areas only to flee again becoming an IDP.^32^ Therefore, the concept of displacement needs to be framed as evolving. Governments and humanitarian actors tend to prioritise immediate short-term emergencies and underestimate the long-term needs.^33^ This leads to gaps in provision for IDPs, even after their permanent resettlement.

By placing water access within the broader context of spatial justice and the evolving concept of displacement, this paper seeks to examine how repeated displacement intersects with spatial inequalities. It argues that IDP resettlement is not simply a logistical challenge but a deeply political process that reinforces patterns of exclusion. Through these lenses, the paper critically explores how humanitarian and governmental actors engage with, and at times reproduce the marginalisation of IDPs through their interventions.

## 3. Methods

### 3.1 Study location

This study was conducted in Hargeisa, the capital city of the Republic of Somaliland, which is internationally recognised as part of Somalia but self-governing since declaring independence in 1991.

In 2021, Somaliland was home to an estimated 601 850 IDPs out of a total population of 6 million.^34^ Recurrent displacement, driven by a combination of wars, droughts and economic instability,^35^ has further placed pressure on authorities, significantly limiting their capacity to provide adequate protection and essential services to displaced populations.

In Hargeisa, IDPs account for approximately ten percent of the total population of 1.2 million.^36^ This places a considerable strain on already limited urban infrastructure, especially public services like water. Water is supplied to the city by HWA using an infrastructure originally designed in 1974 for a population of 175 000.^37^ Although the network capacity and production have been significantly expanded through government and donor investment,^37^ the system remains unable to meet the growing demand driven by the high birth rates, the return of the diaspora and the influx of IDPs.^38,39^ As a result, less than half the city is connected to the water network^40^ and informal service modalities and small-scale independent water businesses play a significant role in water delivery.^41^

IDPs live across 20 informal settlements, commonly known as IDP camps by local authorities. A government agency, NDRA, is responsible for IDPs. However, they often rely heavily on support from international actors due to limited financial and institutional capacity.^42^

Water trucking (Fig. 1d) is the most visible and widespread form; it is unregulated, and prices fluctuate based on fuel costs, distance, and seasonal changes.^41^ Another form of water provision comes from water kiosks, which are small outlets connected to Hargeisa Water Agency's (HWA's) pipeline (Fig. 1c). Kiosk operators purchase water from HWA and sell it for a small profit and serve as intermediaries, making water accessible in smaller quantities to households, donkey cart operators (Fig. 1b), and small motor trucks (Fig. 1a) who transport water to final users, particularly in densely populated areas, with no road access. For IDPs, these informal services are the main means of water access.

**Fig. 1:**
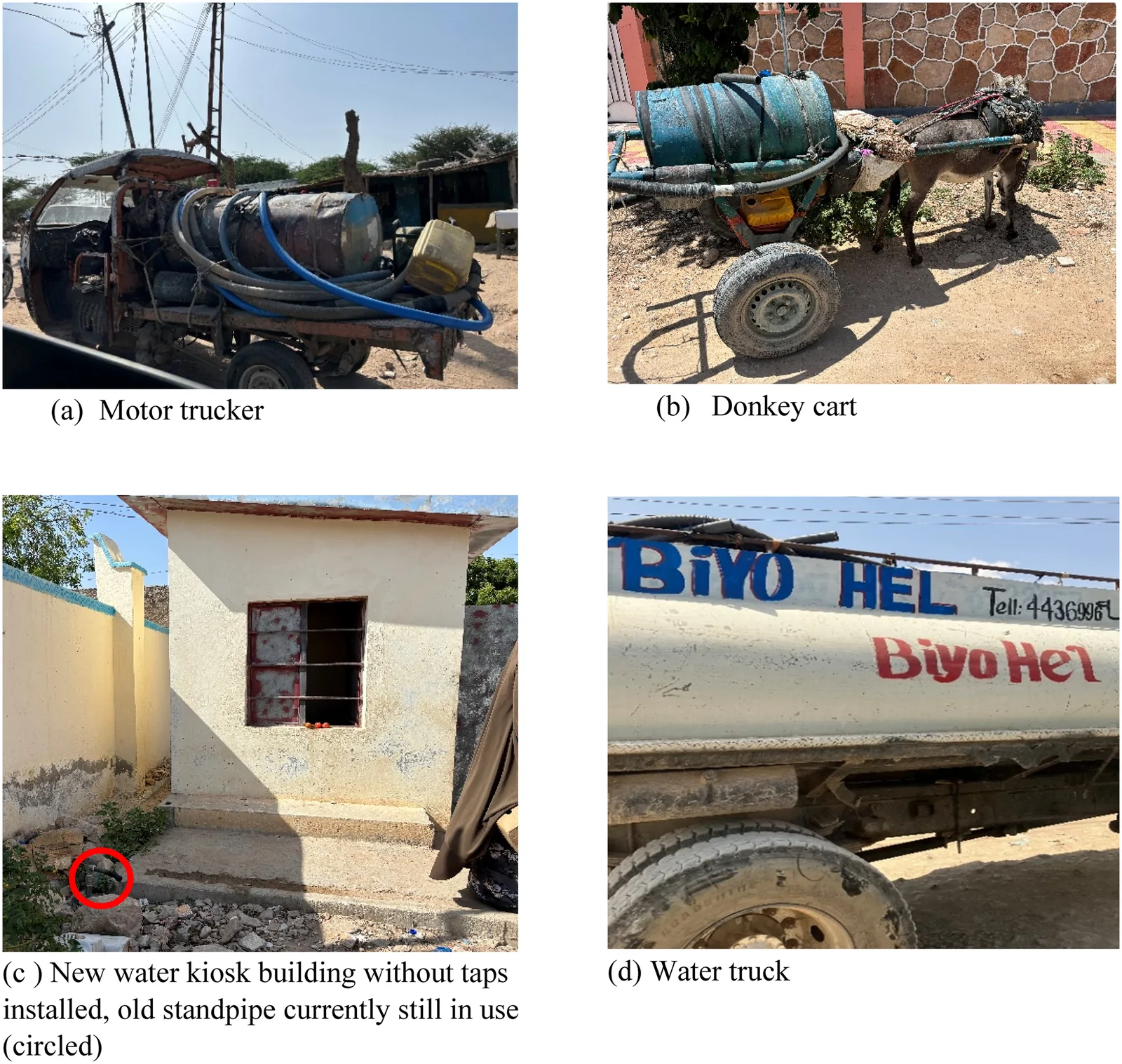
Informal service providers, showing (a) motor trucker, (b) donkey cart, (c) new water kiosk building without taps installed, old standpipe currently still in use (circled), and (d) water truck.

A key issue is the resettlement of IDPs living on private land targeted for urban development or in settlements deemed at risk. The majority of IDPs arrived in Hargeisa following the civil war in the 90s, while others are more recent arrivals displaced by recurrent droughts.^35^ A significant portion of these IDPs have been displaced multiple times, either from other regions of Somaliland or within Hargeisa itself. Many have self-settled on private land in the hopes of eventually being resettled by the government. However, the situation is made worse as landowners push for IDP eviction in order to develop their land. Land in Somaliland, particularly in urban areas is a valuable economic asset.^43–45^ The rapid and unplanned expansion of the city has led to severe overcrowding.^46^ As a result, the availability of land within the city is extremely limited as most of the urban space has already been developed.^47^ In many areas, people extend their properties onto roads, beyond official boundaries. All this combined has made it increasingly difficult to resettle IDPs within the city itself.

### 3.2 Data collection

The first stage involved a scoping review, which included a period of familiarisation (scoping review) where key stakeholders were identified. In the second stage, six IDP sites (see Fig. 2) were selected to cover a range of characteristics from each of these criteria:

**Fig. 2:**
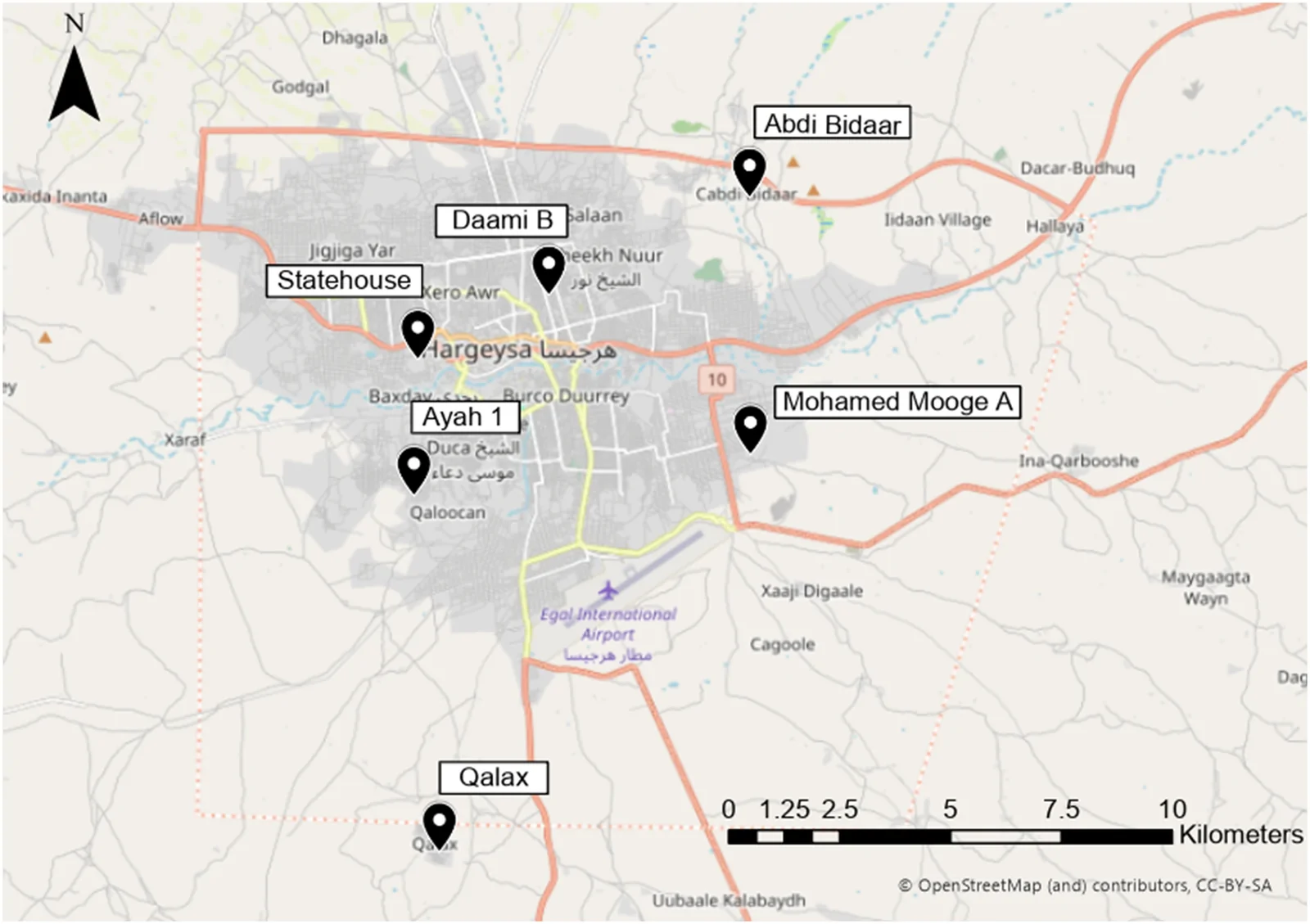
Map showing the six IDP sites included in this study.

1. Sociodemographic background: in two out of the six sites, the IDPs are from a marginalised caste group Gabooye*.*^48^ Across the remaining four sites, the IDPs are from a mix of majority clans.

2. Geographical location: the distance from Waaheen Market, the central marketplace, was used to define this. Three sites were outer (>5 km), two inner (<3 km) and one middle (3–5 km).^49^

3. Population density: one site is very densely populated (>10 000 people per km^2^), four out of the six sites were moderately to densely populated (3000–10000 people per km^2^) and one site was low to moderately populated (1000–3000 people per km^2^).

4. Access to services: services such as transportation, healthcare and education. Three sites with good access (<500 m), one with fair access (500 m–1 km), and two with poor access (>1 km) were selected.^50^

5. Land ownership: IDPs across four out of the six sites own their land, one site is on private land and one site on government land. Land titles are typically issued in these resettlement cases, but only Somali nationals are legally permitted to hold formal ownership documents. Refugees from other backgrounds cannot own land under national regulations and therefore do not receive land titles. As a result, land ownership in this context reflects a specific policy arrangement for Somali IDPs rather than a criterion for displacement status.

The final six sites (Statehouse, Mohamed Mooge A, Ayah 1, Qalax, Daami B and Abdi Bidaar) reflect a range of conditions and context within the wider landscape in Hargeisa.

Given the sensitive nature of this study involving IDPs who may have encountered traumatic events, several steps were taken to ensure the privacy and dignity of the study participants. Prior to data collection, ethical approval was obtained from the Loughborough University Ethical Review Sub-Committee (Project ID: 2024-14183-18538). Interviews with all participants were only conducted after obtaining written informed consent, and on the basis that they would be terminated if any distress or discomfort was felt or demonstrated by the participant.

A total of 30 interviews were conducted across three stakeholder groups. Ten participants from government, NFPs and NGOs were interviewed, together with 14 interviews with informal water providers and six interviews with IDP committee leaders between May and June 2024.

Those providing support to IDPs were recruited through purposive sampling. Many of these were identified during the initial scoping visit with additional contacts sourced through snowball sampling *via* referrals from IDP committee leaders and local networks. Interviews continued until the research team observed that participants' responses were becoming repetitive across stakeholder roles. This pragmatic stopping point was subsequently validated during formal thematic analysis, which confirmed that no new substantive themes emerged beyond the data collected.^51^

The first author conducted all interviews at participants' places of work with meeting times and locations arranged in advance. All the interviews were in English, semi-structured and lasted between 30 minutes and 1.5 hours.

The IDP committee leaders' recruitment was conducted in collaboration with The National Displacement and Refugee Agency (NDRA) who facilitated access by notifying the committee leaders in advance of the visit, coordinating a suitable time and date. In cases where the committee leaders were unavailable, the deputy leader who could convey the overall state of their site was interviewed. To gather comprehensive insights, the first author conducted semi-structured interviews using open-ended questions with follow-up questions based on the participants' responses. An interview guide was designed according to Braun and Clarke^51^ beginning with general questions and progressing to more specific questions. The interviews were conducted in Somali and lasted between 20 and 50 minutes.

Informal service providers were approached randomly in and around the six selected IDP sites, where they were observed operating during transect walks. In total, fourteen interviews were carried out with informal service providers to provide a comprehensive overview and to capture a range of perspective on the challenges of water delivery. This included two water kiosk owners, three water truckers, five members of the water trucking committee, two donkey cart operators, and two motor truck drivers (see Fig. 1). Initial interviews with younger truckers yielded limited insights, leading to follow-up interviews with senior committee members.

The research team comprised of two data collectors who were present for all interviews with the committee leaders and the informal service providers; the interviews with those providing support was conducted by the first author only.

The data collected for this study is part of a more comprehensive study examining water access and displacement. However, this study focuses mainly on support provided from the perspectives of those providing it in the six selected sites. This approach was chosen to gain deeper insights and better contextualise the dynamics of support and its effectiveness. Interviews were selected as the method to allow for a more detailed understanding of the complexities involved in providing potable water provision.

### 3.3 Data analysis

Interviews were audio-recorded, transcribed, and translated from Somali to English where necessary. The resulting transcripts were analysed thematically using NVivo software, following the six-step approach outlined by Braun and Clarke:^51^ familiarisation, coding, generating themes, reviewing themes, defining and naming themes, and producing the final report. The analysis began with a thorough familiarisation with interview transcripts. Using an inductive coding approach, the first author conducted initial coding directly from the data using the interview guides as a framework. Using NVivo (2020), the coding process was repeated, resulting in an extensive list of codes (*n* = 70). The codes were grouped to capture broader concepts, and subsequently four themes were created.

To examine how the views differed between IDPs and those providing support to them, we conducted co-occurrence analysis, comparing how often each theme was raised by each group.

## 4. Results

### 4.1 Participant characteristics

The roles of those providing support varied widely from founders to project officers (Table 1). The length of time participants had been in their position ranged from 2.5 months to over 10 years. The demographic profiles of the IDP committee leaders consisted of mainly males in their 40s–50s; only one female deputy leader was interviewed. The demographic profile of the IDP committee leaders was predominantly male, with only one female deputy leader interviewed. This gender imbalance reflected the composition of formal committee leadership across the selected IDP sites rather than a deliberate sampling decision, as leadership positions were predominantly held by men. Women's representation in these formal leadership structures remains limited, reflecting broader gendered social and institutional barriers to women's participation in community decision-making.

**Table 1:** Participant demographics

Participant ID	Gender	Age group	Role	Number of years in role
WK1	F	40–49	Water kiosk owner	14
WK2	F	50–59	Water kiosk owner	20
WT1	M	18–29	Water trucker	6
WT2	M	18–29	Water trucker	4
WT3	M	40–49	Water trucker	3
WT4	M	50–59	Water trucker committee member	16
WT5	M	40–49	Water trucker committee member	20
WT6	M	50–59	Water trucker committee member	22
WT7	M	50–59	Water trucker committee member	21
WT8	M	50–59	Water trucker committee member	20
MT1	M	60–69	Motor trucker	30
MT2	M	18–29	Motor trucker	4
DC1	M	18–29	Donkey cart owner	2.5
DC2	M	18–29	Donkey cart owner	2
CL1	M	40–49	Committee leader	2
CL2	M	40–49	Committee leader	2
CL3	F	50–59	Committee leader	2
CL4	M	40–49	Committee leader	2
CL5	M	40–49	Committee leader	2
CL6	M	50–59	Committee leader	1
KI1	M	30–39	Project engineer	2.5
KI2	M	30–39	Project officer	6
KI3	M	40–49	Director of IDP	7
KI4	F	20–29	Camp coordinator	2
KI5	M	30–39	Program coordinator	5 months
KI6	F	40–49	Senior camp coordination and camp management (CCCM) officer	4
KI7	M	40–49	Security officer	2.5
KI8	M	30–39	Country representative	3
KI9	F	40–49	Consultant	2.5 months
KI10	F	80–89	Founder	22

The water truckers interviewed at the start of the data collection tended to be younger, aged 19 or 20, with 3–6 years of experience. These younger truckers were employed by the truck owners and did not manage the operations themselves. Generally, their responses were brief and lacked awareness of broader issues. When asked about challenges they faced in their day-to-day operations, most did not report any difficulties. However, one trucker mentioned a committee that led to further inquiry and an interview with a committee member, followed by a group interview with four additional committee members. These committee members were all males and in their late 50s and provided a more comprehensive understanding of the barriers and governance issues impacting water access.

### 4.2 Interview themes

The following themes summarise the key insights from the qualitative interviews and are grouped into four themes. The first theme, explores how the resettlement of IDPs offers security through land ownership but comes with new forms of exclusion, including access to water services. The second theme reflects the disconnect of organisations and how they often fail to respond to the needs of IDPs. The third theme examines how the formalisation of water systems disrupts the livelihoods of informal service providers. The fourth theme concerns the limited autonomy of IDP committees. The following sections present the four themes identified through the analysis, with illustrative participant quotations used to provide evidence for the identified patterns and to illuminate participants' perspectives.

**Theme 1:** resettlement is perceived to improve long-term security through land ownership but creates spatial vulnerabilities that reduce IDPs' access to affordable and reliable water services.

Resettlement to peripheral areas provides IDPs with greater land security, but isolates from economic opportunities and essential services previously accessible in urban centres. Water access dominated IDP committee leaders' accounts of resettlement, raised in 19 mentions across their interviews, compared to just two mentions amongst those providing support, who instead emphasised land ownership as the primary outcome of resettlement. This suggests a mismatch between institutional understandings of successful resettlement and IDPs' priorities. At Abdi Bidar and Qalax, both committee leaders reported that after relocation they now have fewer essential services. This is evident in their accounts of irregular water delivery: “*we are far from the city, water trucks come to us sometimes*” (CL4), and dependence on donations: “*I swear to God it is hard, some families get trucked water and resell amongst themselves for 2000 shillings, when they don't get the donated water*” (CL6).

Access to water varied considerably across the six sites. The two peripheral resettlement sites (>5 km from the central market area) relied exclusively on water trucking, whereas the inner-city sites (<3 km from the central market area) had access to multiple sources, including HWA connections, kiosks, motor trucks and donkey carts. At Daami B, the committee leader highlighted the financial barriers to accessing cheaper alternative: “*water trucks and donkey carts bring us water... we want taps, but we don't have the funds to connect to the pipeline*” (CL3). At Statehouse, the committee leader also implied that more affordable solutions are needed: “*the cost of water is a major problem, other sites get free water*” (CL1). The site on private land, Mooge A, (3–5 km from the central market) was entirely dependent on external support for water. The committee leader here described this as a lifeline and emphasised that if this limited support was withdrawn, the community would be left with nothing: “*we have a water truck that has brought us water for the past 6 years. Once or twice a week… People are struggling a lot now, if they cut off the necessities or they stop we don't have anything*” (CL2). NGOs reported prioritising this site over others, “*identified as the most vulnerable*” (KI6) as they risk eviction. Peripheral resettlement therefore increased dependence on intermittent water trucking while reducing access to smaller, more affordable and diverse water-service options.

The most concerning challenges reported after resettlement also included the physical distance from water sources, the lack of adequate storage tanks and having to purchase in higher quantities: “*where we get water from is very far, people do not have somewhere to pour water [water tanks], the smallest quantity that is sold is five barrels and people cannot afford this*” (CL4).

Water insecurity also extended to perceived water quality. Self-reported water quality concerns were particularly pronounced during the rainy season, when many participants described relying on the river as a supplementary water source. These rivers are normally dry but become accessible when it rains, prompting households to use them for washing, bathing, and sometimes drinking water.^52^ CL2 also reported that water trucks themselves were, at times, sourced from open water sources. These accounts indicate that limited access to reliable water services can increase reliance on sources perceived to present quality concerns.

Sanitation also remains an unresolved challenge in the newly established sites. Although some progress has been made with the delivery of latrines, these efforts are not in line with the needs of the population. CL6 highlighted that all households need toilets but only thirty had been built.

The IDPs residing on private land and awaiting resettlement have a long history of displacement as the committee leader noted: “*14 years ago we were relocated from Digaale, the ones that remained settled here*” (CL2). These IDPs cannot construct permanent sanitation facilities, resulting in open defecation. The committee leader here imagines the resettlement will be a turning point, one that will bring them improved infrastructure and dignity: “*we are waiting to be resettled to a better place where we can have toilets*” (CL2). However, this aspiration is undermined by ongoing land disputes. As the committee leader elaborates: “*people own this land, always asking us to free up their land… land is sold above our heads, which puts us in a difficult situation*” (CL2). An NGO assisting IDPs at this site also emphasised that securing land is critical: “*the services are missing; they have been living there for a long time now they have been forgotten, number one is getting them land, when they get land, the rest will come*” (KI6).

The government's resettlement policies focus on allocating land over establishing sustainable infrastructure. Participant KI7 explained that the government have authority to “*reclaim up to 30% of privately owned land for public use such as hospitals, police stations, schools, mosques, or anything that comes under public interest*”. This policy primarily targets large private landowners. The resettlement process also reflects a symbolic importance of land ownership. For example, the settlement Abdi Bidar is named after the individual who “donated” the land. Authorities push land ownership as progress, emphasising the transition from “*camps*” to “*settlements*” (KI5). Participant KI7 further remarked, “*Yes, they are better off. They have their own land, schools, access to buses, and better air quality*”. The officer explained that before relocating, IDPs were “*living on someone's land for 17 years, the owner constantly asked for them to be moved from his land, the IDPs wanted somewhere they owned… they had dogs released on them, burnt, shot at, abused, they were happy to be resettled they were kissing the sand [land] they were given*” (KI7). This act of kissing the land symbolises gratitude, much like prostration in Islam where worshippers bow before God. Other committee leaders expressed gratitude. Responses were often accompanied with a common expression used to show gratitude to God: “*Alhamdullilah*” (praise be to God) and the belief in God's provision, one stating “*we get nothing else except what God provides*” (CL2).

The resettlement is challenging, as an NGO worker highlighted that, it involves a team of NGOs with funding to assist the IDPs, and the government's contribution is limited: “*they get a team of NGOs with money, they resettle them with this money, and the government has a small contribution to this*”. The worker elaborates that rather than providing land, the government pressures citizens to give up their land, framing it as a public good “*but the land they are taken to is not government land. For example, you have land now, and the government asks you to give this up for the greater good or public interest, so it is not an easy process*” (KI7).

The municipality also focus on large-scale, one-day relocations: “*on the relocation day we involve the police, and get 100 trucks for the materials and buses for the IDPs. We must relocate them in one day, otherwise the whole of Hargeisa would come*” (KI6). This underscores the intense demand for land not just from IDPs but also from many other city residents.

**Theme 2:** international organisations fail to address local needs due to centralisation and disconnection despite strong coordination among themselves.

International organisations, including the UN, often fail to effectively address local needs due to their centralisation and disconnect from the communities they aim to serve. The resulting frustration stemming from this situation is evident. One individual, a founder of a non-profit with an affiliated NGO and a retired UN worker explained that the head offices of organisations are in Mogadishu as Somaliland is not recognised and that: “*many will say we only do what the head office tells us to do*”. She goes on to say “*it's like having a tractor with a broken part and the spare part is somewhere else… take your machine where it can be fixed or it can be of some use. You can hear it from my voice that I am not very happy about what is being done. I am not happy about what is being done for displaced people. In camps they are given hope, they sit there waiting for a miracle to respond to their needs. They need food, water, medication, education and shelter. If you cannot give me that, I don't need you to be in my country… I know this is their job, this is their mandate, and they are not doing their job in Somaliland, and someone needs to challenge them*” (KI10). Rather than fulfilling their mandate “*their presence is protected*” and justified by “*local employment (including watchmen, cleaners, drivers) and large property rentals*” (KI4).

This frustration is also highlighted with decisions and interventions made for IDPs. Interventions often focus on short-term solutions, rather than developing durable systems mainly due to funding. A project engineer noted, “*we cover their basic needs, immediate relief... based on the emergency trucking standard of WASH, the Sphere standard of 7.5 litres per person per day*” (KI1). Another project officer similarly explained that not all needs are covered “*it is likely that we can only cover 20%*” and “*depends on how we get funding*” (KI2). Furthermore, the reduction in funding from donors has led to reduced operations. For example, one NGO's support was reduced from 19 sites to just 4, despite unchanged levels of need. As one senior CCCM officer explained: “*donor priorities changed... we were asked to identify the most vulnerable people, and these four sites have the greatest need*” (KI6).

No IDP committee leaders explicitly discussed the internal coordination of international organisations. In contrast, all those supporting IDPs mentioned this at least twice, and raised issues relating to decision-making and funding constraints.

At Qalax, a few facilities including a school and a free healthcare facility have been constructed for the community. However, the healthcare facility was reported to be largely unused. An NGO worker conducting environmental health checks at the school explained that IDPs “*who did not attend the school would come with their children for examination*” (KI9). They did so because they “*saw a doctor present*” and offering “*free checks*”. She noted: “*there are health centres right next to where they are, but for some reason they don't access them... There is some sort of barrier there and we haven't gotten to the bottom of it*” (KI9). The IDPs not utilising the free healthcare facility shows communication gaps and highlights how funds can be deployed in ways that fail to align with community needs.

Despite these disconnects, there have been positive examples of coordination between government and NGOs. For example: “*we hold regular coordination meetings such as the monthly CCCM cluster coordination meetings*” (KI3). When NGOs were asked about their collaborations with government and other organisations, many highlighted their efforts to engage with the government. One participant emphasised the good communication between themselves and NDRA, noting: “*there are meetings with the government, NDRA is our line ministry. If a project comes, we go to NDRA, if they have meetings we take part, so our communication is good. We also undertake cluster meetings, CCCM with NDRA, livelihoods meetings*” (KI4). This sentiment was echoed by others who highlighted their collaborations with the government: “*we work with several government bodies, the municipality, ministry of employment and social affairs, ministry of health*” (KI2).

Collaborations also include advocacy; participant K15 stated that they were in contact with the municipality and supported with the relocation. He further explained that they advocate to the Ministry of Water, HWA and development partners. In addition to direct government collaborations, the coordination extends to specific sites and interventions; he explains that they work with the government, and they classify the areas of work*.* He further adds that “*if one organisation is working for Qalax and implement such WASH infrastructure, the government refer other organisations with similar projects to other areas*” (KI5). Another emphasised the role of NDRA: “*NDRA is the one that links all of us, I remember when they were relocating a camp they called all the NGOs that work in the camps and they were telling them each one has to pick what they can do for the camp, for example one organisation dug 60 latrines and some gave cash transfers*” (KI8).

**Theme 3:** expansion of formal water systems threatens informal service providers' livelihoods.

HWA have been expanding their capacity in phases, with the first phase seeing the number of households connected almost doubling from 21 000 in 2018 to 41 000 households in 2024. While further expansions are planned, these achievements coexist with persistent service gaps. The increases in water distribution infrastructure outpace the available bulk water supply, leading to shortages in areas previously served. HWA cannot expend services to the entire city due to its limited scope and leaves a significant portion of the population dependent on alternative water sources.

The impacts of formal water expansion on informal provision were raised by all informal service providers and three IDP committee leaders; none of the organisations providing support raised this as an issue. Informal providers made 36 explicit references to declining demand, storage restrictions, rising operating costs, reduced profitability, and the threat posed by continued formal expansion, while IDP committee leaders made ten references linking informal providers to continued water access, particularly where formal connections were unavailable or unaffordable.

Informal service providers fill the gaps left by formal water infrastructure, and the recent expansions of water supply have disrupted these services, undermined the livelihoods of service providers as well as impacted those who depend on them. Water kiosk operators, for example, now face strict bans on storage and a limit on the number of days they operate. One operator explained that they previously sold water everyday but now sell every three days: “*before [the expansion] was better for me, the reason being I had [storage] tanks and would sell water every day. They have built me this little kiosk but it hasn't benefited me, it's only a house, but the water situation was better before*” (WK1).

Motor truckers also report growing operational constraints tied to new water distribution regulations: “*every area has a day they get piped water. I might get water from the Pepsi area today, but I cannot get it from there tomorrow, even if it's running,*” (MT1). He added that these rules force them to travel to distant areas to refill, increasing both time and fuel costs: “*before, kiosk owners had tanks and we used to buy water from the same kiosks daily. Now they are not allowed tanks, those seen with tanks get fined*” (MT1). Another motor trucker explained that his ability to sell water is constrained by established customer relationships in different areas: “*they do not know me in that area*” (MT2), he highlights how truckers can typically operate within a fixed area where they are recognised and have a customer base.

Increased competition has further reduced the viability of informal trucking. A water trucking committee member (WT6) described a saturated market. He shared that they used to supply six loads a day to a hotel, but now the hotel has its own truck. As profitability declines, many providers have left the sector altogether. One motor trucker (MT2) explained that the first phase of the water expansion led many potential truckers to reconsider careers, while many already in the business stopped. He also expressed concern that if the expansion continues, his job could be at risk.

A kiosk operator in Daami (WK2) described the resulting collapse in demand and the impacts on their livelihoods*:* “*I do not even sell a cubic meter of water, people all have piped water. Maybe in three days, the amount I sell doesn't even reach a cubic meter... You see that house over there, I sell to them but all those houses have piped water. I may have one or two houses that come with a jerrycan every two days. There is no water to sell… In the 20 years I have been working, I have less business now. The people in this area all have water*” (WK2).

These disruptions extend beyond livelihood losses, directly affect displaced communities. Informal providers, particularly motor truckers, donkey carts, and kiosks enable small quantities of water to be purchased, and many allow for delayed payment options and buying on credit: “*if people cannot pay, we ask them when they can and we go*” (WT3), options not offered by HWA who require payments the month after usage and non-payment resulting in disconnection of service, plus additional reconnection fees.

**Theme 4:** committee leaders act as essential intermediaries but have limited autonomy, and logistical constraints restrict their ability to advocate effectively for their communities.

All six committee leaders described their role constraints at least three times, reflecting a broader lack of agency within IDP communities to self-organise or determine their own representation. Committee leaders are selected and governed through processes managed by NDRA: “*we facilitate elections every 2 years*” (KI3). When questioned about the resettlement, IDP committee leaders mentioned that the decisions were solely the government's decision and responses underscored an absence of choice: “*we were told to settle here*” (CL4).

A committee leader explained that they have restricted ability to act on behalf of their communities: “*I can't target organisations myself. NDRA needs to be aware, we work with organisations NDRA bring to us*” (CL1). This reliance on NDRA was further emphasised by CL2: “*our first point of contact is always NDRA*”. Another committee leader explained the unequal power dynamics and their relationship with formal authorities: “*Only when he [NDRA IDP Director] has time for us, when he wants. We have no open door. How we reach him is difficult*” (CL5). Even opportunities for collaboration are dictated by NDRA, as noted by another committee leader “*NDRA tell us to go to seminars organised by NGOs, we meet them there with other committee leaders and talk*” (CL2). This also extends to projects and resource allocation, as another participant emphasised: “*they [NGOs] decide what projects to fund based on their priorities, not ours*” (CL4).

Another voiced frustration is the silenced local priorities and urgent concerns raised by committee leaders remaining unaddressed, such as sanitation shortages: “*10 households share one toilet*” (CL1). Similarly at another site, a committee leader stated that “*water is a government responsibility, they need to reduce the costs for connecting*” (CL3). The lack of follow through was also mentioned with IDPs being left without clear answers: “w*e have spoken to the organisation that have been helping us, we think they haven't started yet but have told us that they are coming*” (CL6).

In addition to structural exclusion, committee leaders report a lack of institutional respect, with meetings frequently delayed*:* “*organisations come to interview me, they make appointments with us and show up three hours late… they shouldn't be eating up my time and show up when we planned to meet… they can't expect us to wait three hours*” (CL1).

NDRA maintain regular communications with the IDP committees: “we *regularly meet with the committees, we go to the sites, we have good communication with them*”. Apart from NDRA, only one other organisation (K18) spoke positively about its relationship with committee leaders and the supportive approaches they take. This organisation described its relationship by saying they are in contact with the committee at each site and meet with them when the distribute water, they also described the use of WhatsApp groups to share information and weekly direct calls. In contrast, other organisations took a more direct, top-down approach, often exercising authority. This was reflected in their language where committees were often positioned as passive recipients. For example, they spoke about providing: “*WASH training to the committees*” (KI2), and “*we trained them*… *we also taught them good hand washing, and we left hand sanitisers*” (KI9). Committee leaders (CL2, CL3 and CL5) expressed the need for a more supportive approach to address the ongoing needs.

## 5. Discussion

This study identified the misalignment between many actors, including international organisations and the lived realities of IDPs themselves. Interventions are centralised and often implemented with no consultation with IDPs. This echoes existing critiques of international organisations and government actors and the minimal input of end users and missed opportunities for effective solutions.^53^ It exacerbates insecurity, fosters resentment, and erodes trust in humanitarian efforts.^54^ A more inclusive approach would allow for better implementation strategies and help develop attitudes towards ownership. Currently, the water support being provided to IDPs does not align with their needs; many expressed needing affordable alternatives to water trucking but implementing such solutions remain unfeasible in isolated sites where HWA does not plan to extend its network. Somaliland's political status, being unrecognised by many countries, means that many organisational decision making hubs and head offices are based outside of the country and this sometimes constrains the ability to respond to the specific needs of IDPs.

In the resettlement of IDPs, government actors often treated land ownership as an indicator of long-term security, whereas IDP committee leaders placed greater emphasis on access to water and other essential services. This reveals a trade-off between tenure security and service accessibility. In the two peripheral resettlement sites examined, water access was limited to trucking, while inner-city sites had access to multiple water-service options. The contribution therefore extends beyond confirming that displacement creates water-access difficulties and shows how planned peripheral resettlement can itself reproduce water insecurity by relocating IDPs beyond the reach of formal infrastructure.

Since 2007, over 8 000 households have been resettled across 11 sites; some earlier resettlements are in a more inner-city location and provided IDPs with permanent houses through the UN-Habitat's Permanent Shelter Programme.^55^ Those resettled between 2007 and 2015 through this programme are considered assimilated by the government and live among the host population; despite this, they are still included in official government records and receive support although less than in their initial phase of displacement. Those relocated more recently in 2021 and 2024, although they own land, do not have permanent housing, and their shelter is constructed of corrugated iron sheets. These IDPs are also in isolated areas without a nearby hosting community. As a result, they continue to receive more targeted support. Principle 29.1 of the Guiding Principles stipulates that those resettling must not suffer discrimination as a result of having been displaced and shall have the right to participate fully and equally in public affairs and have equal access to public services.^9^ Mooney^56^ proposed three overlapping criteria for determining what signals the end of displacement. The first, cause-based criteria focused on whether the root causes of displacement have changed. The second, solution-based criteria focused on voluntary return or resettlement with full rights and protections. The third, needs-based criteria argued displacement ends when IDPs no longer have specific vulnerabilities or needs related to their displacement.

Planned resettlement efforts discussed in the existing literature focus on reducing physical exposure to environmental hazards, overlooking the broader dimensions.^57^ In many contexts, governments depend on international organisations for financial and technical assistance in planning and executing resettlements.^58^ This support is often fragmented, or poorly coordinated and lacks accountability and contributes to poor decision-making.^5,12^ NGOs and governments frequently operate independently, resulting in overlapping efforts and repeatedly targeting the same communities with similar forms of assistance.^12^ However, in the context of this research, the findings show that there are good connections between the government and international organisations demonstrated by sharing of project information, especially in the lead up to resettlement, holding multiple meetings and ensuring the resettlement day goes smoothly.

Government led resettlement schemes can result in secondary displacement where IDPs move out of new resettlement sites.^59^ Similar to Miller *et al.*'s^59^ research in the Mekong Delta, Vietnam, the Hargeisa case highlights how environmental resettlement intended to reduce vulnerabilities often expose people to new risks such as declining livelihood opportunities resulting in a rise in outmigration. A key driver of this secondary displacement is the spatial injustices, with the new sites often disconnected from labour markets. The experience of the committee leader who chose to self-settle on private land 14 years ago from another IDP site demonstrates this repeated displacement. Already experienced two displacements, he now awaits a third resettlement, the new site likely to be in an isolated area, reproducing the spatial injustices described earlier. Their imagined futures are tied to the prospect of receiving land, which they see as a pathway to stability. Land is deeply rooted in the societal values of the communities that hold it, shaping identity, belonging and social status beyond its purely economic worth.^60^ This makes land registration a highly bureaucratic and often contentious process, particularly for resettled IDPs who are issued land titles as a means of formalising their claims and integrating them into the existing system.

This study also sheds light on the increasingly uncertain position of informal service providers whose livelihoods are threatened as a direct consequence of formal water system expansions. In the first phase of the water expansion, the bulk water supply has significantly increased and has offered improvements in access with areas that have been reliant solely on water trucking now able to receive water through HWA at a lower rate. However, this expansion has introduced challenges in distributing water across the system and has also increased intermittency due to pressure imbalances. The completion of additional phases of the expansion project, which includes the addition of four reservoirs placed across four of the five districts of Hargeisa, will allow for a more controlled and equitable distributions compared to the current where households closest to the current reservoir enjoy better access. Previous research has examined formalisation approaches^61^ and the regulation of informal providers, and efforts to integrate them into formal market systems.^62^ Ahlers *et al.*^62^ show that formalisation in Greater Maputo, Mozambique imposed bureaucracy and financial burdens that excluded small-scale independent water providers (SSIPs). In addition to this, formalisation also resulted in cost rises for consumers. Similarly, Boakye-Ansah *et al.*^61^ reveal how Kenyan partnerships with informal providers legitimised exploitative practices, as utilities focused on revenue over service quality and leaving low-income consumers worse off. This study has further shown that in the context of displacement in Hargeisa, formalisation efforts not only disrupt service reliability for existing connected customers through increased supply intermittency, but also the livelihoods of informal service providers and their customers. Informal service providers, particularly motor truckers reported being pushed out of the market, similar to Maputo, are not meaningfully integrated into the new system, instead they are excluded without alternative support. However, larger informal providers, like water truckers in Hargeisa, are adapting differently; rather than disappearing, some reported to be seeking work from HWA. This mirrors the situation in Maputo, where organised and capitalised larger providers found ways to benefit from formalisation. These findings strongly align with the perspectives in the existing literature, demonstrating that formalisation can exacerbates systemic weaknesses, such as reinforcing hierarchies, failing to accommodate smaller providers and undermine livelihoods.

Moreover, by drawing on the perspectives of IDP committee leaders, this study provides a nuanced analysis of their roles within the broader governance framework. While IDP committee leaders are often positioned as key decision makers, this study reveals the significant limitations on their autonomy and influence over decisions. These include their restricted ability to act independently on behalf of their community, their reliance on NDRA and organisations exercising authority over them, resulting in their roles only being symbolic and used primarily for channels of communication. There are formal procedures that exist but in practice, decisions are made through informal meetings and negotiations. Trust, leadership and shared understanding amongst committee leaders and government officials are essential for relationship building. This finding aligns with previous research that highlights how leadership is frequently undermined and lack real authority.^63^ In this study, committee leaders discussed the support they received in broad terms and their problems with collaborating with organisations more specifically. While some channels of support exist, they still reflect broader patterns of exclusion. Committee leaders reported facing logistical challenges, such as lack of transportation. Organisations regularly run training for committee leaders away from the IDP sites and assume everyone can get to them. In addition, the lack of direct communication channels with those providing support limits their ability to advocate effectively for their communities. These limitations reinforce patterns of exclusion and agency, where those most affected by decisions are often least empowered to influence them.^64^ By paying closer attention to IDP needs and by viewing committee leaders as key assets, more adaptive and sustainable solutions can be designed.

Two challenges emerged in this research. First, there is limited long-term infrastructure planning for water in resettlement sites located beyond the foreseeable reach of the piped network, creating a persistent form of spatial inequality. In practice, this leaves resettled IDPs dependent on intermittent and often more costly water delivery, while transferring the continuing financial and logistical burden of provision to government and humanitarian actors. It may also increase exposure to reliability and perceived water-quality concerns where alternative or open water sources are used. Addressing this requires water-service planning to be integrated into resettlement decisions from the outset. Where extension of the piped network is not technically or financially feasible, government and water authorities should identify and commit to context-appropriate permanent alternatives, with clear arrangements for financing, operation and maintenance. From a spatial justice perspective, providing secure land without a credible pathway to essential infrastructure risks institutionalising rather than resolving displacement-related disadvantage.

Second, the resettlement process itself excludes IDPs and they are resettled without consultation and simply instructed to move. Moss *et al.*^65^ stress the importance of engaging a broad range of stakeholders in the resettlement process. In the absence of inclusive partnerships and greater accountability from international organisations, planned resettlement efforts risk reinforcing the very vulnerabilities they aim to address. Additionally, without active engagement from IDPs in the planning and execution of resettlement and service provision, these initiatives are likely to overlook their actual needs, leading to dissatisfaction. At both of the resettlement sites, the committee leaders expressed dissatisfaction and voiced strong discontent with the conditions and support available in their new locations. This mirrors experiences from resettlement programmes in Nigeria^66^ and Sinhalese IDPs in Sri Lanka.^67^

Since data collection has been completed, a new government has taken office in December 2024 and has made changes to several of the leaderships of various ministries and government agencies. One of the major shifts includes turning the NDRA from an agency into the Ministry of Resettlement and Humanitarian Affairs (MORHA). This may significantly raise the profile of IDPs within the government and lead to more long-term planning, more government funding and the stronger integration of IDPs into the national development plans. However, there is also a risk that the original humanitarian and protection focus of the NDRA could be reduced if the ministry becomes overly bureaucratic or politically driven.

Many of the issues discussed in this paper, including the consequences of formalisation of water systems, the agency of IDPs, the misalignment between priorities and IDP needs and the resettlement of IDPs to peripheral areas of cities, are not new; these have been brought into sharper focus by ongoing resettlement initiatives and changes in support. The new sites are frequently marked by inadequate infrastructure and limited-service provision and place IDPs in precarious and invisible positions. It highlights the need for more coherent frameworks that recognise the unique challenges faced by IDPs, while ensuring their active participation in shaping solutions that directly impact their lives, thereby contributing to more effective, sustainable appropriate interventions.

## 6. Conclusions

This study identifies key dynamics, including the loss of livelihoods of informal water providers, the limited influence of IDP committee leaders, the centralisation of decision-making processes, and policies emphasising land ownership over basic service provision. It was found that those resettled often faced greater challenges related to both their livelihoods and access to water compared to their previous sites. These challenges largely stem from the absence of water infrastructure policies in managing long-term services for IDPs. Current policies tend to focus on land allocation and physical resettlement, assuming that land ownership equates to stability. This focus, however, neglects critical aspects of service provision. As a result, IDPs remain in a state of dependency on both the government and international actors, with little assurance that basic infrastructure such as a water network will ever reach them. These issues, when analysed through the perspectives of IDP committee leaders, informal service providers, and government and humanitarian actors, offer a multifaceted understanding of the barriers and governance dynamics undermining water access for IDPs.

The findings from this study should be interpreted considering the limitations. These include those providing support not reflecting the full range of local and international organisations operating in Hargeisa, which may limit the comprehensiveness of the insights gathered. This was primarily due to practical constrains including limited time and resources and conducting fieldwork overseas. While the study provides valuable insights, including a broader range of stakeholders could have added further depth. The participant sample was predominately Somali males, and only seven out of the 30 interviews were with females. This may limit fully capturing gender specific perspectives and needs. Future studies should aim to include a more diverse representation of organisations and genders to better capture a variety of perspectives. Additionally, most of the participants providing support to IDPs had been in their roles for less than three years, with two having been in their roles for less than six months. This is partially due to short term recruitment contracts commonly offered by organisations, often aligned with the duration of specific projects. While all the key informants contributed valuable insights, future studies should consider individuals with more experience in the field that are able to speak more on the long-term trends or institutional dynamics. Finally, one limitation of our study is that sampling was facilitated by NDRA, which could have potentially introduced bias by directing towards more cooperative individuals. As a result, some voices may have been excluded due to this gatekeeping. Future research should aim to include more diverse selection of participants to better understand the full spectrum of perspectives.

Subsequent research should also focus on determining effective strategies to enhance the participation of IDPs, particularly their committees, in the planning and implementation of resettlement and water services, ensuring interventions are aligned with evolving needs. Additionally, further studies could investigate the extent to which political constraints, such as the lack of international recognition of Somaliland, affect NGOs' operational responses. Furthermore, a mixed-method approach to investigate the topic would be useful, combining survey disaggregated data with in-depth interviews can provide representative insights. Noting that the connection between IDP resettlement and service provision has received relatively limited attention, while this study adds insights by providing a richer and more nuanced understanding particularly with IDP resettlement in Hargeisa, further research should specifically account for the political constraints shaping water access post resettlement and longitudinal studies to examine how water access evolves over time. Addressing these gaps in future research could help provide a more holistic understanding of the challenges and opportunities.

## Conflicts of interest

No interests to declare.

## Supplementary Material

EW-OLF-D6EW00002A-s001

EW-OLF-D6EW00002A-s002

## Data Availability

The data supporting this article have been included as part of the supplementary information (SI). The interview guides and codebook supporting this article are provided in the SI. See DOI: https://doi.org/10.1039/d6ew00002a.
