# Supplementary material for: Resettlement and spatial injustices: governance and water access challenges for IDPs in Hargeisa, Somaliland
Source: Environ Sci (Camb). 2026 Oct 6. Online ahead of print. doi: 10.1039/d6ew00002a (PMC13715719; doi:10.1039/d6ew00002a)
Supplement: Supplementary file 1 [file EW-OLF-D6EW00002A-s002.pdf]

This journal is © The Royal Society of Chemistry

Interview guides

Interview guide for IDP committee leaders

Interviews will be semi-structured and will be themed around community dynamics (relationships within the community) and water service provision and support received from NGOs and government.

1. What is your age?
2. How long have you lived at this site?
3. How many people are part of your household?
4. What is your occupation?
5. What is role is as an IDP community leader?
6. How would you describe the current state of water availability and safely managed sanitation services in your settlement?
7. What are the main barriers encountered in ensuring universal and equitable access to water and sanitation services?
8. Can you please describe the type and nature of support, specifically related to WASH provision you receive from government or NGOs, if any?
9. Can you share examples of partnerships/ collaborations between your community and organisations that have influenced positive changes related to water access/affordability?
10. Can you share examples of any problems and difficulties when collaborating with organisations?
11. Please describe the level of support you receive for improved WASH. How does it compare with what other settlements receive?
12. What improvements or interventions do you think could improve water service provision in your settlement?
13. How do you currently engage with your community members to identify their water needs and priorities?
14. How do you address concerns or feedback from community members regarding water access?
15. What challenges/conflicts if any, do you face with the host community in terms of access to water resources?
16. Please describe the relationship that exists between the IDP community and the host community.
17. What are the key challenges and gaps that need to be addressed in relation to WASH?
18. Is there anything else you would like to add concerning WASH services in the IDP camp?

Interview guide for those providing support

Interviews will be semi-structured and will be themed around meeting the needs of IDPs, the support provided and how this matches their needs.

1. What is your current role?
2. How long have you worked with your organisation?
3. Do you work directly or indirectly with IDPs?
4. How many IDP settlements are you currently working with?
5. What support does your organisation provide to IDPs, specifically related to WASH?
6. How often does your organisation provide this support? Is support scheduled, or are they responsive to emerging needs?
7. To what extent do you think the WASH needs of IDPs are being met in these settlements?
8. Can you share strategies/ initiatives your organisation has undertaken to influence policy changes aimed at improving the access/ affordability of water for IDPs?
9. How does your organisation facilitate and encourage community participation in relation to services or programs you provide?
10. How does your organisation work with local communities, governments, and other stakeholders?
11. Is there anything else you would like to add concerning WASH services in the IDP camps?

Interview guide for informal service providers

Interviews will be semi-structured and will be themed around the role informal water providers play.

1. What is your age?
2. How long have you been working as a service provider?
3. Where do you get your water from? Do you use multiple sources?
4. Can you tell me about the areas of Hargeisa you currently cover?
5. Can you tell me about how people would contact you for water services?
6. On average, how many households do you serve daily? How many of these households are living in IDPs sites?
7. How do you set your price for water? Is pricing based on location or units purchased?
8. What challenges do you come across when delivering water to households?
9. How do you perceive your role as a service provider in addressing water issues within the communities you serve?
10. Can you tell me about any collaborations between yourself and local authorities/ organisations aimed at addressing water issues?
11. Is there anything else you would like to add concerning provision of WASH services in the IDP camp?
